# An Exploratory Study on the Microbiome of Northern and Southern Populations of *Ixodes scapularis* Ticks Predicts Changes and Unique Bacterial Interactions

**DOI:** 10.3390/pathogens11020130

**Published:** 2022-01-21

**Authors:** Deepak Kumar, Latoyia P. Downs, Abdulsalam Adegoke, Erika Machtinger, Kelly Oggenfuss, Richard S. Ostfeld, Monica Embers, Shahid Karim

**Affiliations:** 1 School of Biological, Environmental and Earth Sciences, The University of Southern Mississippi, Hattiesburg, MS 39406, USA; Deepak.Kumar@usm.edu (D.K.); Latoyia.Downs@usm.edu (L.P.D.); Abdulsalam.Adegoke@usm.edu (A.A.); 2 Department of Entomology, Pennsylvania State University, University Park, PA 16802, USA; etm10@psu.edu; 3 Cary Institute of Ecosystem Studies, Millbrook, NY 12542, USA; oggenfussk@caryinstitute.org (K.O.); ostfeldr@caryinstitute.org (R.S.O.); 4 Division of Immunology, Tulane National Primate Research Center, 18703 Three Rivers Rd., Covington, LA 70433, USA; members@tulane.edu; 5 Center for Molecular and Cellular Biosciences, University of Southern Mississippi, Hattiesburg, MS 39406, USA

**Keywords:** *Ixodes scapularis*, microbiome, salivary glands, midgut, ovaries, 16S rRNA sequencing, *Borrelia burgdorferi*, *Rickettsia buchneri*

## Abstract

The black-legged tick (*Ixodes scapularis*) is the primary vector of *Borrelia burgdorferi*, the causative agent of Lyme disease in North America. However, the prevalence of Lyme borreliosis is clustered around the Northern States of the United States of America. This study utilized a metagenomic sequencing approach to compare the microbial communities residing within *Ix. scapularis* populations from northern and southern geographic locations in the USA. Using a SparCC network construction model, we performed potential interactions between members of the microbial communities from *Borrelia burgdorferi*–infected tissues of unfed and blood-fed ticks. A significant difference in bacterial composition and diversity was found between northern and southern tick populations. The network analysis predicted a potential antagonistic interaction between endosymbiont *Rickettsia buchneri* and *Borrelia burgdorferi* sensu lato. The network analysis, as expected, predicted significant positive and negative microbial interactions in ticks from these geographic regions, with the genus *Rickettsia*, *Francisella*, and *Borreliella* playing an essential role in the identified clusters. Interactions between *Rickettsia buchneri* and *Borrelia burgdorferi* sensu lato need more validation and understanding. Understanding the interplay between the microbiome and tick-borne pathogens within tick vectors may pave the way for new strategies to prevent tick-borne infections.

## 1. Introduction

Vector-borne diseases affect over one billion people every year and have been expanding alarmingly in recent years [1,2,3]. Among vector-borne diseases, Lyme disease is one of the most reported infectious diseases in the United States and corresponds to over 90% of vector-borne infections in North America [4,5]. Almost 300,000 cases of Lyme disease are reported every year in the United States, and *Ixodes scapularis* is known as its primary vector. Most Lyme disease cases are clustered in Northeastern and Upper Midwest States [6]. *Ix. scapularis* ticks are also prevalent in the Southern States, but the dearth of Lyme disease infections is linked with the restricted distribution and scarcity of the primary *B. burgdorferi* s.l. reservoir, namely white-footed mice, *Peromyscus leucopus* [7]. For *B. burgdorferi* s.l., an infection rate of ~35–50% has been reported among ticks from the Northeastern states [8,9], whereas tick populations in the Southern states are more rarely infected [10,11,12,13,14].

Several studies have reported the microbial composition residing within ticks [15,16,17,18,19,20,21,22]. Despite this work, limited information exists on which to base comparisons of the microbiome residing within *Ix. scapularis* from the Lyme disease endemic and non-endemic areas or between different tissue types within ticks. Moreover, there is a dearth of information on the interaction between recently identified tick endosymbionts or commensal microorganisms and *B. burgdorferi* s.l. or pathogenic microbes. Efforts have been made to identify commensal and symbiotic microbes in the tick microbiome [8,23]. Significant variations associated with geography, species, and sex in *Ixodes* microbiota have been reported [14]. The impact of microbiome communities on physiological processes in ixodid ticks, including reproductive fitness and vector competence, has been suggested [24,25,26,27,28,29]. In the United States, tick microbiome studies have shown variation in sex, species, and geography. In Canada, the *Ix. scapularis* microbiome from eastern and southern regions did not differ significantly concerning the geographic origin, sex, or life stages (Clow et al., 2018). These variable results motivate additional studies to generally detect whether geographic and demographic patterns exist.

This study conducted a comparative analysis of the microbiome residing within the *Ix. scapularis* populations collected from Lyme-disease-endemic and -non-endemic areas in the United States, utilizing 16S rRNA sequencing. This study also provided an insight into the microbiome composition in unfed and partially blood-fed tick tissues, including midgut, salivary glands, and ovaries.

## 2. Results

### 2.1. Illumina MiSeq 16S V1–V3 Sequencing

Sequencing reads were acquired from a limited amount of tick samples (39 single ticks and 15 tick tissues). A minimum of three biological replicates for each sample and tissue type was used. The total number of reads obtained for whole-tick samples collected from Louisiana, New York, Oklahoma, and Pennsylvania was 2,427,191, corresponding to 2812 operational taxonomic units (OTUs), the minimum number of reads for a sample was 36,358, and the maximum number of reads for a sample was 114,478. For tick tissues, the total number of reads was 546,024, corresponding to 1009 OTUs; the maximum number of reads for a sample was 56,785; and the minimum number of reads was 17,258. In our analyses, each tissue sample was rarefied to 5000 sequences. In comparison, whole-tick samples were rarefied to 16,000 sequences. The rarefaction curve (Supplementary Figure S1) plotted between the number of observed OTUs and the number of sequences, as mentioned above, reached plateau, suggesting sufficient sample coverage for further analysis.

### 2.2. Comparative Analysis of Bacterial Communities Residing within Ix. scapularis

We observed substantial variation in bacterial profiles in tick populations from Louisiana, New York, Oklahoma, and Pennsylvania. Phyla Actinobacteria, Proteobacteria, Firmicutes, and Bacteroidetes cover for >90% reads for female ticks from these populations (Supplementary Table S1). Proteobacteria were found to be the dominant phylum among all female tick populations, excluding the female Pennsylvania population, in which Bacteroidetes were dominant. A relative abundance of 36% Actinobacteria was present in female Oklahoma ticks, but in other female ticks, its coverage was substantially low as 0.58%, 6.13%, and 3.45% in Pennsylvania, Louisiana, and New York, respectively (Supplementary Table S1). Interestingly, female Pennsylvania ticks showed 60% relative abundance of Bacteroidetes, whereas this phylum was substantially less well represented in females from Oklahoma, New York, and Louisiana, at 0.07%, 7.5% and 0.49%, respectively (Supplementary Table S1). In male ticks, a significantly high level of Actinobacteria (88%) was found in Oklahoma ticks, and levels were dramatically lower in Louisiana and New York males, at 3.5% and 10%, respectively (Supplementary Table S1). New York male ticks were not included in this study, due to unavailability. The level of Proteobacteria was also found to be much lower in Oklahoma males (6.19%) than Louisiana males (94.03%) and Pennsylvania males (50%) (Supplementary Table S1). Bacteroidetes (29%) and Tenericutes (10.4%) were substantially higher in Pennsylvania males than other whole unfed male ticks, Oklahoma males (0.26%-Bacteroidetes), and Louisiana males (1.49%-Bacteroidetes), while Tenericutes were rare in other male ticks (Supplementary Table S1).

**Figure 1:**
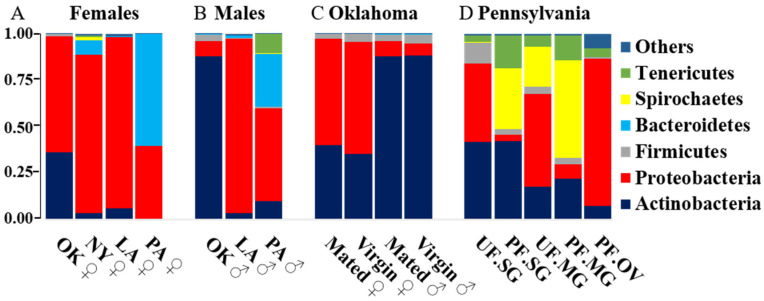

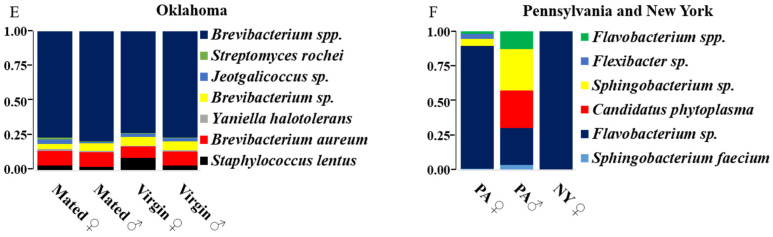
Bacterial profiling and relative abundance in *Ixodes scapularis* ticks at phylum level (**A**) among female ticks and (**B**) male ticks from Louisiana (LA), Pennsylvania (PA), Oklahoma (OK) (**C**), and between virgin and mated ticks from Oklahoma (**D**) in unfed and partially fed tick tissues collected from Pennsylvania (PA). (**E**) At species level in Oklahoma ticks and ** (**F**) Pennsylvania ticks. ** If ticks from a particular location have shown significant difference in relative bacterial abundance at phylum level, then they have further been shown at species level.

The bacterial profiling of mated and unmated tick populations was similar at the phylum level (Figure 1A–C). Among these ticks, the dominant phylum in both males and females were Actinobacteria (~88%) and Proteobacteria (~56 to 60%), respectively (Figure 1A,B). Nonetheless, both male and female Oklahoma ticks contained ~2 to 5% Firmicutes, but other tick populations (NY and PA) contained either <1% or negligible Firmicutes (Figure 1A,B). Further analysis of Oklahoma ticks at species level showed Firmicutes’ presence, including *Jeotgalicoccus* sp. and *Staphylococcus lentus* (Figure 1E). At the same time, Actinobacteria included *Brevibacterium aureum*, *Yaniella halotolerans*, *Brevibacterium* sp., and *Streptomyces rochei* species (Figure 1E). As expected, both New York and Pennsylvania tick populations were infected with a significantly higher percentage of Spirochetes, ranging from 2% reads in New York (♀) and 0.14% in Pennsylvania (♂) respectively (Figure 1A). The prevalence of *B. burgdorferi* s. l. in *Ix. scapularis* ticks from New York and Pennsylvania ranges from 40 to 70% [8,9]. All the ticks and tick tissues, excluding the ovary, were infected with *B. burgdorferi* s.l. The results presented here are a comparison of percentage *B. burgdorferi* s.l. reads among all microbial reads in the sample. For example, ticks from Pennsylvania and New York are *B. burgdorferi* s.l.–infected, and 16S rRNA sequencing yielded a total of 100 reads from these ticks. Among those 100 reads, 2 and 0.14 reads belong to *B. burgdorferi* s.l. from New York and Pennsylvania ticks, respectively. The rest of the reads belong to other microbes residing within these samples.

The Pennsylvania male and female ticks contained ~60% and 28.8% Bacteroidetes, respectively, and 7.48% in female ticks from New York (Figure 1A,B). Bacteroidetes included *Sphingobacterium faecium*, *Flavobacterium* sp., *Sphingobacterium* sp., *Flexibacter* sp. and *Flavobacter* spp. (Figure 1F). However, spirochetes were absent in both Louisiana and Oklahoma tick populations, and only ~1.49% reads with Bacteroidetes were detected in the Louisiana female ticks (Figure 1 and Supplementary Table S1).

**Figure 2:**
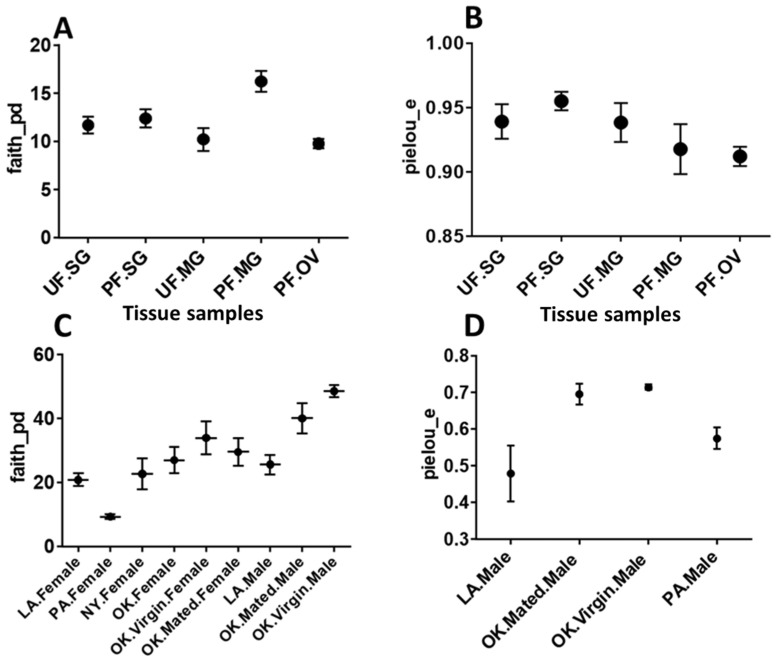
Comparisons of bacterial alpha diversity among tick tissue samples (**A**,**B**) and ticks collected from different geographical locations (**C**,**D**). Plotted data represent alpha-diversity based on Faith’s phylogenetic distance (faith_pd) demonstrating relative richness among bacterial communities, while Pielou’s index represent the evenness of the bacterial community among different samples. Upon using the Benjamin and Hochberg correction, we found that all the adjusted-*p*-values (q-values) were insignificant. UF—unfed, PF—partially fed, SG—salivary gland, MG—midgut, OV—ovary, LA—Louisiana, PA—Pennsylvania, NY—New York, OK—Oklahoma.

Differences and variations were also noted in bacterial compositions of New York and Pennsylvania female tick populations. Proteobacteria (86%) was the dominant phylum in New York female tick populations, whereas Bacteroidetes (60%) was dominant phylum in Pennsylvania female ticks (Figure 1A). Bacterial profiling of the New York female and the Oklahoma female ticks were significantly different at both phyla and species level (Figure 2 and Supplementary Table S2 species-level profiling). The Oklahoma female ticks contained 36.3%, 62.32%, 1.23%, and 0.06% reads of phylum Actinobacteria, Proteobacteria, Firmicutes, Bacteroidetes, and Spirochetes, respectively. However, the New York female ticks harbor 3.45%, 85.75%, 0.28%, 7.48% and 1.96% reads of Actinobacteria, Proteobacteria, Firmicutes, Bacteroidetes and Spirochaetes. Interestingly, both New York and Oklahoma tick populations at the species level demonstrated considerable differences in their bacterial profiling and abundance (Supplementary Table S2). Specifically, bacterial species such as *Brevibacterium* spp. (OK ♀ = 46.5%, NY ♀ = 0.33%), *Pseudomonas lurida* (OK ♀ = 0.17%, NY ♀ = 23.4%), *Brevindumonas diminuta* (OK ♀ = 0.05%, NY ♀= 8.42%), *Rickettsia* spp. (OK ♀ = 29.23%, NY ♀ = 15.8%), *Flavobacterium* spp. (OK ♀ = 0.04%, NY ♀ = 6.62%), *Stenotrophomonas rhizophila* (OK ♀ = 0.0358, NY ♀ = 14.52%), *Pseudomonas fluorescens* (OK ♀ = 0.04%, NY ♀ = 8.16%), and *Brevibacterium aureum* (OK ♀ = 6.22%. NY ♀ = 0.04%) demonstrated substantial differences in bacterial profiling between these female ticks collected from different geographical regions, specifically the Northeastern and Southern United States.

### 2.3. Bacterial Composition of Unfed and Partially Engorged Tick Tissues from Pennsylvania (PA)

An important difference in phylum-level microbial profiling was detected among different tissues collected from unfed and partially blood-fed female tick tissues (Figure 1D, Supplementary Table S3). There was substantial difference in relative abundance of Proteobacteria (UF.SG = 42.27%, PF.SG = 3.43%), Firmicutes (UF.SG = 11.18%, PF.SG = 3.29%) and Spirochetes (UF.SG = 0.26%, PF.SG = 32.78%) between unfed (UF) and partially fed (PF) salivary gland (SG) tissues (Figure 1D, Supplementary Table S3). In midgut (MG) tissues, the trend for Proteobacteria (UF.MG = 50.34%, PF.MG = 7.7%) and Spirochete abundance (UF.MG = 21.6%, PF.MG = 52.7%) was similar to that of the salivary gland (SG) (Figure 1D and Supplementary Table S3). Unfed ovary was not available for this work. In the case of the partially fed ovary (PF.OV), the level of Proteobacteria (85.6%) was considerably higher than other tissues, while the Spirochete level (PF.OV = 0.12%) was substantially low (Figure 1D and Supplementary Table S3). It was noted that Bacteroidetes were the most dominant (relative abundance, 60%) phylum of Pennsylvania female ticks at the unfed-whole-tick level but were completely absent at the tissue level (SG, MG, and OV).

A considerable difference in microbial profiling at the phylum level was detected among different tissues collected from the Pennsylvania unfed and partially blood-fed female tick tissues (Figure 1D). Unfed salivary glands contained 42%, 42.27%, and ~11.2%, respectively, of Actinobacteria, Proteobacteria, and Firmicutes, whereas partially blood-fed salivary glands harbored 42% Actinobacteria reads, followed by a substantially reduced level of Proteobacteria (3.43%) and Firmicutes (3.2%). Interestingly, both Spirochetes and Tenericutes levels increased from 0.26% to 32.77% and 3.37% to 17.66%, respectively (Figure 1D and Supplementary Table S3). Supplementary Table S3 shows the bacterial species abundance in tick tissues. Unfed midgut contained 50.34%, 21.61%, and 17.5% Proteobacteria, Spirochetes, and Actinobacteria, respectively; while partially engorged midgut of Proteobacteria decreases substantially to 7.7%, Spirochetes increases drastically to 52.7%. The unfed ovary was unavailable, and the partially engorged ovary contained 85.56% of Proteobacteria, 8.11% of Actinobacteria, 5.24% of Tenericutes, and <1% of Firmicutes and Spirochetes (Supplementary Table S3).

### 2.4. Presence of Rickettsia Buchneri in Partially Fed Tick Tissues and Pattern of Likely Competitive Interactions with B. burgdorferi s.l.

**Figure 3:**
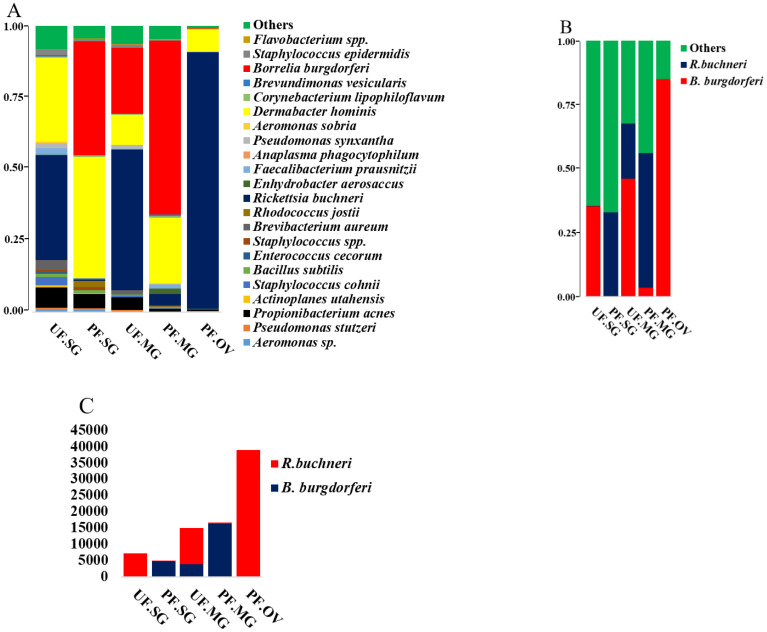
Microbial profiling at (**A**) species level in unfed and partially fed tick tissues (SG, MG, and OV). These field-collected *Ixodes scapularis* ticks were collected from Pennsylvania. (**B**) Probable competitive interaction between endosymbiont *Rickettsia buchneri* and *Borrelia burgdorferi.* (**C**) Absolute abundance (mean of the number of sequences of biological replicates) of *Borrelia burgdorferi* and *Rickettsia buchneri* in tick tissues from Pennsylvania (PA). UF—unfed, PF—partially fed, SG—salivary gland, MG—midgut, OV—ovary.

*R. buchneri* reads in partially blood-fed salivary glands decreased drastically to 0.33% compared to unfed salivary glands (35.38%); however, levels of *B. burgdorferi* s.l. increased from 0.23% to 32.77% (Figure 3A). Similarly, partially engorged midgut tissues also showed the same trend as salivary glands, i.e., the level of *Borrelia* increased substantially, and the rickettsia level decreased substantially (Figure 3B and Supplementary Table S4). Interestingly, the partially fed ovaries showed ~85% reads of rickettsia and as expected, 0.12% of *Borrelia*, since it is not vertically transmitted to the next generation (Figure 3B and Supplementary Table S4). These results demonstrated the infection of *R. buchneri* in the midgut (UF/PF: 46%/4%) and salivary glands (UF/PF: 35%/0.3%), and these levels highlight the substantial reduction in the level of *R. buchneri* upon blood feeding. The mean absolute abundances of *B. burgdorferi* s.l. (UF.SG = 21.67, PF.SG = 4820.67, UF.MG = 3875.33, PF.MG = 16,284.70, PF.OV = 54) and *R. buchneri* (UF.SG = 7154.3, PF.SG = 44, UF.MG = 11,044.67, PF.MG = 292.67, PF.OV = 38,828) also indicate the same trend as that of their relative abundances (Figure 3C and Supplementary Table S5).

### 2.5. Diversity Analysis

Kruskal–Wallis non-parametric tests were performed to determine the effects of tick geographic location (LA, PA, NY, and OK) on α-diversity metrics, using QIIME 2. Faith_pd-diversity index, *p*-value, and adjusted-*p*-value (q-value) between the samples are as follows: Louisiana and Pennsylvania female ticks (H = 5.4, *p*-value = 0.02, q-value = 0.101), Louisiana male ticks and Oklahoma mated male ticks (H = 3.85, *p*-value = 0.049, q-value = 0.101), New York and Pennsylvania male and female ticks (H = 5.4, *p*-value = 0.02, q-value = 0.101), Oklahoma and Pennsylvania female ticks (H = 5.4, *p*-value = 0.02, q-value = 0.101), and Oklahoma and Pennsylvania virgin male (H = 4.5, *p*-value = 0.0338, q-value = 0.101) (Figure 2C). Pennsylvania ticks were phylogenetically the least rich, while Oklahoma virgin males were the richest in bacterial diversity among samples studied here. At the tissue level, in the midgut from partially engorged ticks, we found the richest in diversity and ovaries were the least rich in bacterial diversity (Figure 2A). Tissue samples did not demonstrate statistically significant evenness (pielou_e) in bacterial diversity (Figure 2B). Mated and virgin Oklahoma male ticks contained similar bacterial profiles (Figure 2D).

**Figure 4:**
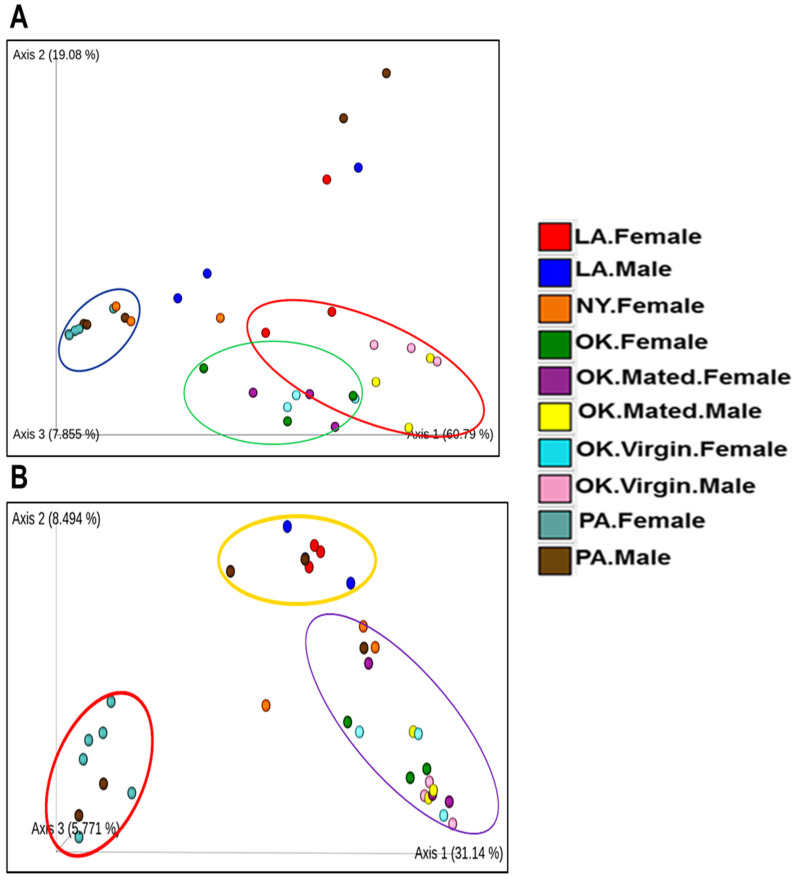
PCoA plot for male and female ticks collected from OK, LA, NY, and PA. (**A**) Weighted UniFrac distance, the bacterial composition in female from Pennsylvania (PA.Female), New York (NY.Female) and male from Pennsylvania cluster together. (**B**) Unweighted UniFrac plot.

**Figure 5:**
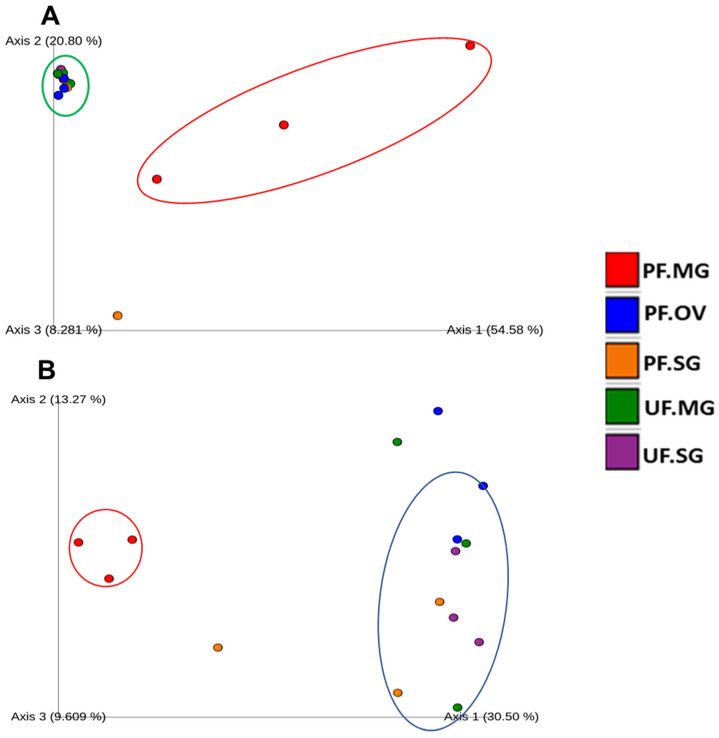
PCOA plot for unfed and partially fed tick tissues collected from Pennsylvania. (**A**) Weighted UniFrac and (**B**) unweighted UniFrac. UF—unfed, PF—partially fed, SG—salivary gland, MG—midgut, OV—ovary.

As it is clear from the Principal Coordinate Analysis (PCoA) plot of unweighted UniFrac distances, most of the tick samples from different locations (NY, OK, PA, and LA) contained different bacterial profiles (*p* = 0.001), as determined by PERMANOVA (Permutational multiple analysis of variance). The PCoA of unweighted UniFrac distances of bacterial communities showed that the first two axes (Axis1 and Axis2) explained 31.14% and 8.5% of the variation in the data respectively (Figure 4B). PERMANOVA analysis of Unweighted UniFrac distances, *p*-values and adjusted *p*-values (q-value) in several of the samples when compared pairwise were as follows Louisiana and Pennsylvania female ticks (pseudo-F = 7.33, *p*-value = 0.011, q-value = 0.095625), Louisiana male and Pennsylvania female ticks (pseudo-F = 6.10, *p*-value = 0.017, q-value = 0.095625), New York female and Pennsylvania female (pseudo-F = 7.19, *p*-value = 0.013, q-value = 0.095625), Oklahoma females and Pennsylvania females (pseudo-F = 10.80, *p*-value = 0.008, q-value = 0.095625), Oklahoma mated females and Pennsylvania males (pseudo-F = 2.99, *p*-value = 0.047, q-value = 0.147), Oklahoma mated males and Pennsylvania males (pseudo-F = 3.20, *p*-value = 0.03, q-value = 0.135), Oklahoma virgin females and Pennsylvania females (pseudo-F = 8.124, *p*-value = 0.017, q-value = 0.095625), and Oklahoma virgin males and Pennsylvania males (pseudo-F = 3.76, *p*-value = 0.027, q-value = 0.135) (Supplementary Table S6). For the unweighted UniFrac of whole-tick samples, Oklahoma tick samples (Mated ♀, Mated ♂, Virgin ♀, Virgin ♂), Louisiana ticks (♂, ♀), and Pennsylvania (♀, ♂) are separate clusters for the most part. Interestingly, it also showed a few outliers, such as Pennsylvania males and New York females (Figure 4A). Unweighted UniFrac PCoA plot (Figure 5B) showed that the partially fed midgut is distinct from unfed midgut at the tissue level. In this case, PCoA of unweighted UniFrac distances of bacterial communities in the first two axes (Axis1 and Axis2) explained 30.5% and 13.27% of the variation in the data. Again, in the weighted UniFrac PCoA plot (Figure 5A) also, the partially fed midgut (PF.MG) clustered more distinctly than the unfed midgut (UF.MG) and other tissues indicating that the dominant bacterial diversity in the partially fed midgut is distinct than other tissues. All other tissues, except the partially fed midgut, clustered together separately, indicating common dominant bacteria.

### 2.6. Microbial Interactions

**Figure 6:**
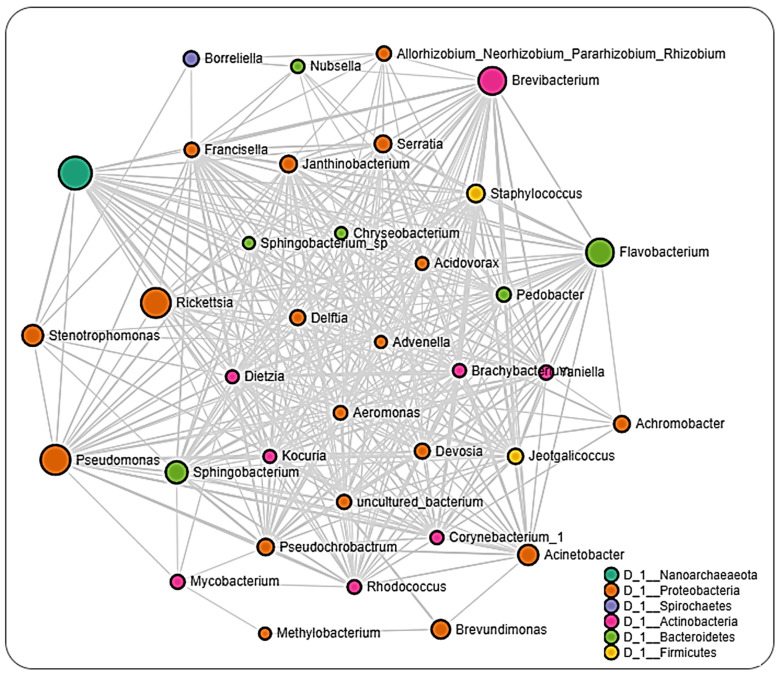
Correlation network analysis. Correlation network maps were generated by using the SparCC approach, with nodes representing taxa at the genus level and edges representing correlations between taxa pairs. Node size correlates to the number of interactions a taxon is involved with. Color-coded legend shows the bacteria phylum each taxon belongs to.

The network analysis of microbial interactions, using the SparCC permutations, revealed 726 interactions amongst 36 taxa when applied to the life-stage dataset from all regions, out of which 358 were positive interactions, while 360 were negative interactions (Figure 6 and Supplementary Table S7). All identified taxa belonged to the phylum Nanoarchaeaeota (Unidentified genus), Proteobacteria (18), Spirochates (1), Actinobacteria (8), Bacteroidetes (6), and Firmicutes (2). Most interactions were seen with the bacteria in the genus *Flavobacterium, Pseudomonas,* and *Brevibacterium*. Bacteria genus from pathogenic group belonging to *Rickettsia* and *Borreliella* were also identified in the network interactions, with *Rickettsia* interacting with more members compared to *Borreliella*.

**Figure 7:**
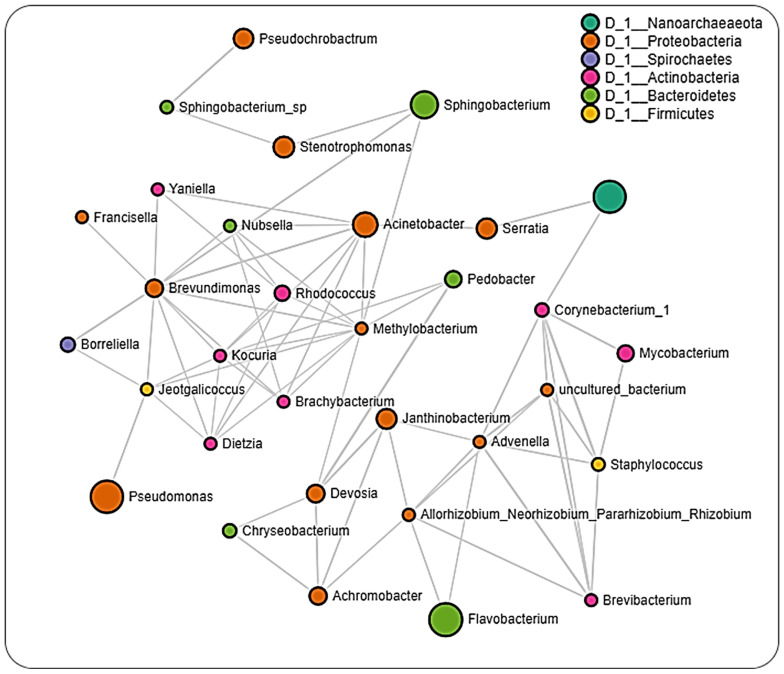
Correlation network analysis across ticks from Pennsylvania. Correlation network maps were generated by using the SparCC approach, with nodes representing taxa at the genus level and edges representing correlations between taxa pairs. Node size correlates to the number of interactions a taxon is involved with. Color-coded legend shows the bacteria phylum each taxon belongs to.

The interaction cluster in which *Rickettsia* was detected revealed several positive and negative correlations with *Francisella*, with the positive correlation being more notable. In the network, *Borreliella* was identified in the same cluster as *Allorhizobium_Neorhizobium_Pararhizobium_Rhizobium*, *Nubsella, Stenotrophomonas,* and notably *Francisella,* all of which were positively correlated to one another. The network analysis performed on datasets from the different regions showed that ticks from Pennsylvania had the most significant partial correlations of 140 involving the most OTUs (32) (Figure 7 and Supplementary Table S8). *Borreliella* was identified across the datasets but was observed to be correlated with different bacterial genera from each region. In the Pennsylvania dataset, *Borreliella* was negatively correlated with *Brevundimonas* and positively correlated with *Jeotgalicoccus* (Supplementary Table S8).

**Figure 8:**
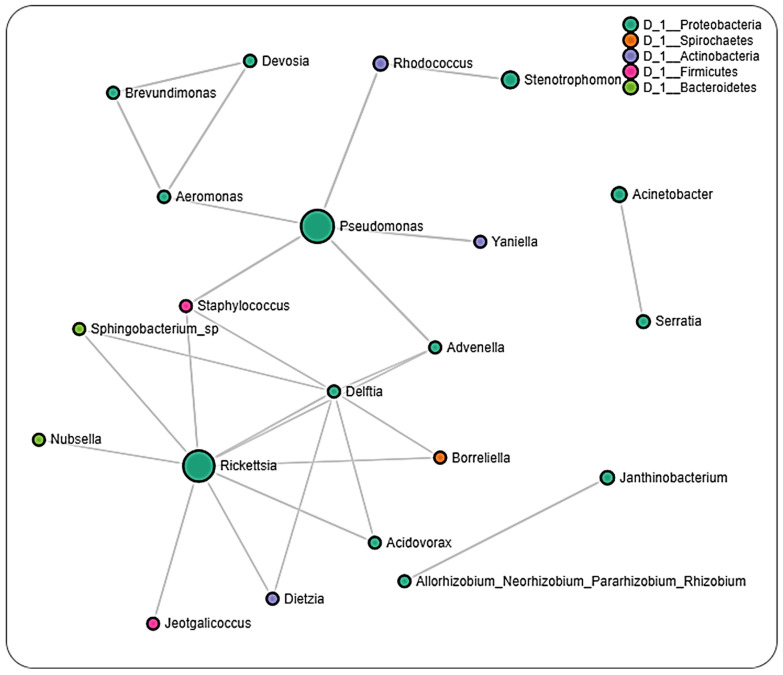
Correlation network analysis across ticks from Louisiana. Correlation network maps generated by using the SparCC approach, with nodes representing taxa at the genus level and edges representing correlations between taxa pairs. Node size correlates to the number of interactions a taxon is involved with. Color-coded legend shows the bacteria phylum each taxon belongs to.

**Figure 9:**
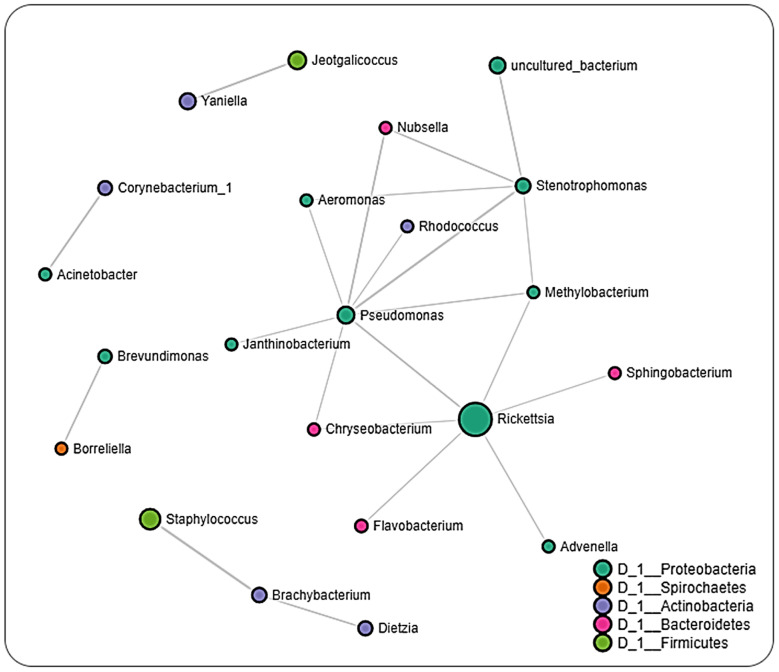
Correlation network analysis across ticks from Oklahoma. Correlation network maps generated by using the SparCC approach, with nodes representing taxa at the genus level and edges representing correlations between taxa pairs. Node size correlates to the number of interactions a taxon is involved with. Color-coded legend shows the bacteria phylum each taxon belongs to.

In ticks from Louisiana, we observed negative partial correlations between *Borreliella* and *Delftia* and positive partial correlations between *Borreliella* and *Rickettsia*, while dataset from Oklahoma only showed partial negative correlation between *Borreliella* and *Brevundimonas* (Figure 8 and Figure 9; Supplementary Tables S9 and S10). We, however, did not detect the presence of *Rickettsia* in the dataset from Pennsylvania ticks. Datasets from Louisiana and Oklahoma both had *Rickettsia* in their network interactions and interestingly share similarities in their partial positive correlations with the genus *Advenella* and *Sphingobacterium*. While datasets from both Oklahoma and Louisiana contained *Borreliella* and *Rickettsia*, a direct significant correlation was only observed between both bacteria in the dataset from Louisiana.

## 3. Discussion

In this study, the microbiome of *Ix. scapularis* from Lyme-disease-endemic (NY and PA) and -non-endemic (LA and OK) regions showed substantial variation by both geography and sex at the organismal and tissue levels. A variation in the microbiota of *Ixodes* ticks with regard to geography and sex has been suggested [14]. Our results are not in complete coherence with other studies [30,31,32,33,34], as those studies were conducted under a variety of different conditions to examine the tick’s microbiome. For example, Sperling et al. [34] analyzed the ticks collected off the cats and dogs from Alberta (Canada), whereas Kwan et al. [31] conducted their studies on *Ixodes pacificus* ticks. Several studies have also identified variation in microbiome among different tick species [35], sexes [36,37], and geographies [14,16]. It remains unclear what factors drive the microbiome’s natural variation [33]. The impact of host and environmental drivers on the microbiome composition is still an under-investigated area [33]. By sequencing DNA from 39 single ticks and 15 individual tissues, our study showed the dominance of Proteobacteria in most of the tick samples except Pennsylvania female and Oklahoma male tick samples. Firmicutes were found only in Oklahoma ticks, and Bacteroidetes only in New York and Pennsylvania ticks. These results raised an important question of whether these ticks in different geographic regions maintain a distinct microbiome and whether these variations among the microbial composition and communities are driven by the host animals or even by soil bacteria [38].

Our data also showed comparatively higher *B. burgdorferi* s.l. reads (~2.5%) in the New York and Pennsylvania female ticks compared to Louisiana and Oklahoma tick populations [4,6]. *B. burgdorferi* s.l. reads were also detected in the Pennsylvania male ticks (Figure 1B), and they were surprisingly higher than other male tick samples. Bacteroidetes were dominant in the Pennsylvania female tick, whereas, in both unfed and partially engorged tick tissues, the level was surprisingly low (Figure 1A,B,D). It suggests bloodmeal-induced changes in the microbiome composition in tick tissues. Interestingly, studies pointed out that vertebrate hosts do not influence the bacterial composition within adult flea and tick species (*Dermacentor variabilis* and *Ix. scapularis*) [35], but blood-feeding in immature developmental stages significantly impacts the bacterial community structuring [39]. Before and immediately after blood feeding of *Ixodes persulcatus* on rats, a report showed similar alpha-diversity but significantly different bacterial profiling of tick [37].

In the present study, the endosymbiont *R. buchneri* was found in the salivary gland, midgut, and ovary, and its level drops significantly when *B. burgdorferi* s.l. multiplies in tick tissues upon blood-feeding (Figure 3B), suggesting a possible competitive interaction that is purely speculative at this point and needs more work to support it. Moreover, the absolute-abundance data of *R. buchneri* and *B. burgdorferi* s.l. (Figure 2 and Supplementary Table S5) support this possible competitive interplay. Recent elegant work from Oliver et al. [40] has shown the growth dynamics of *R. buchneri* in *Ix. scapularis* ticks. This type of competitive interaction has also been demonstrated between *B. burgdorferi* s.l. and gut microbiota in the midgut of *Ix. scapularis* [41] and by *Rickettsia* spp. in the ovary of *Dermacentor andersoni* [42]. The mechanism of co-existence of *B. burgdorferi* s.l. with other microbial pathogens inside ticks remain unknown [43] and is an area of active research. As *B. burgdorferi* s.l. is extracellular and *R. buchneri* is an intracellular microbe, they might not have physical interaction, but they may affect colonization by activating immune and reactive oxygen species pathways [43,44,45]. The endosymbiont *R. buchneri* is also known as “rickettsial endosymbiont of *Ix. scapularis*” [46]. The genome of *R. buchneri* is significantly larger (>2 Mb) than that of pathogenic rickettsiae, and it also has the capability to synthesize vitamin B components, biotin, and de novo folate [46]. As indicated by previous studies, one of the major roles of the symbionts from ticks and other obligate hematophagous arthropods is to provide vitamin B, which that ticks are deficient in, due to their exclusively sanguineous diet [47,48]. The presence of *R. buchneri* was reported to be restricted to tick ovaries [49,50], but a recent study has reported its colonization into tick salivary glands [48]. It might evolve as a pathogen, as it fulfills the prerequisite for an endosymbiont to be transmitted to the vertebrate host by getting colonized into tick’s salivary glands [48]. Our data showed the colonization of this rickettsial species into salivary glands (UF.SG = 35.38%, PF.SG = 0.33%) and the midgut (UF.MG = 46.09%, PF.MG = 3.52%) during the unfed stage, but the level of *R. buchneri* reduces significantly after blood feeding. It is not necessary that this process would lead to a condition of pathogenicity, it might simply be a transmission route to other ticks through co-feeding. Nevertheless, a symbiont in the salivary gland might also be exposed to the host immune system, leading to an antibody response or can interact with pathogens in the salivary gland and may facilitate or impede their transmission [48]. Studies have shown that several genera of bacteria, such as *Rickettsia, Coxiella, Francisella,* and *Midichloria*, persist transtadially and later get transmitted transovarially as a regular process [47,51]. These endosymbionts might remain restricted to the arthropod host and sometimes may be transmitted to the vertebrate host or sometimes may cause disease [47,51]. The distribution of reads for *R. buchneri* in the various tissues is consistent with the rest of the literature [48].

Network analysis across all life-stage datasets from all regions revealed a relatively equal number of positive (~49%) and negative (~51%) interactions to be present. Positive interactions between different bacteria taxa could indicate shared functionality, or even a shared niche within the host organism [52,53,54], whereas negative interactions would point toward an existing or potential competition between bacteria taxa. This would suggest that the microbiome of *Ix. scapularis* favors a balanced distribution between bacteria with potential synergistic and antagonistic interactions. This observation contrasts the detection of greater than 97% positive interactions in the *Ix. ricinus* microbiota, as recently reported by Lejal et al. [55], thus indicating differences exist in the microbial–microbial interaction even between the same tick genera. Most of the interactions observed in the whole dataset were driven by bacteria belonging to non-pathogenic genera, as indicated by the presence of *Flavobacterium, Pseudomonas,* and *Brevibacterium,* further suggesting a contribution of non-symbiotic commensal microbes to the overall microbiome of the *Ix. scapularis* ticks.

Interestingly, we identified OTUs belonging to the pathogenic *Rickettsia* and *Borreliella* genus from the network analysis, with *Rickettsia* observed to interact with more bacteria genera compared to *Borreliella*. A positive correlation was seen to exist between *Francisella* and *Borreliella*. The interaction between *Rickettsia* and *Borreliella* was also observed to vary across different geographical locations. While our network analysis was carried out down to the genus level, the bacterial profile identified the *Rickettsia* identified to the species level as *R. buchneri*, the major endosymbiont of *Ix. scapularis*. The genus *Francisella,* which is endosymbiont, was significantly correlated with *Rickettsia,* suggesting an indication of a co-dependency on two endosymbionts by *Ix. scapularis.* This hypothesis is in accordance with a recent report of a dual endosymbiont dependency observed between *Midichloria* and *Francisella* symbionts in *Hyalomma marginatum* ticks driven by a nutritional adaptation [56]. It has also been shown that, although *R. buchneri* does possess the essential vitamin synthesis genes, some *Ix. scapularis* harbor this endosymbiont that lack these vitamin synthesis pathways, indicating a non-obligatory or facultative endosymbiotic relationship [57,58,59]. This could explain the need to harbor a different class of endosymbiont in *Francisella* that would relieve the exclusive dependence on *R. buchneri,* as seen in this study.

The detection of multiple endosymbiont species has also been identified in other tick species, such as in the *Amblyomma maculatum* tick, although the functional contribution to the tick biology has not been described [60]. *Rickettsia* and *Borreliella* were not identified within the same cluster in the network analysis of the whole dataset; however, both bacteria genera interacted differently in ticks from different locations. A much surprising observation was the presence of *Borreliella* and corresponding absence of *Rickettsia* from the network analysis on datasets of ticks from Pennsylvania. While the small size of the tissue dataset from these regions prevented us from carrying out a network analysis, this observation was further supported by the exclusive presence of *R. buchneri* in unfed salivary glands and partially fed ovarian tissues. A shared presence of both bacteria in partially fed salivary glands and midgut tissue suggests a tissue-driven microbial interaction. This observation contrasts with the report of Aivelo et al., who reported positive correlations between Lyme borreliois *Borrelia* group, *Borrelia miyamotoi,* and *Rickettsiella* in the sister tick *Ix. ricinus,* suggesting a specific interaction dependent on the host tick specie.

## 4. Materials and Methods

### 4.1. Ticks

**Figure 10:**
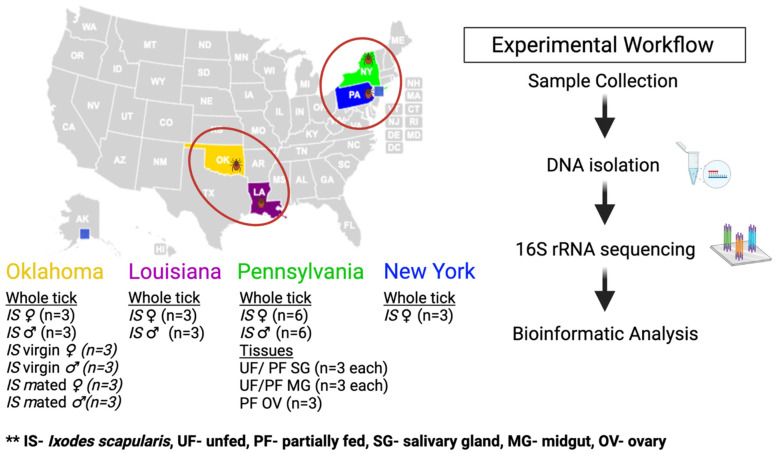
Schematic diagram of the microbiome study of the black-legged tick (*Ixodes scapularis*).

Only adult ticks were field collected from Louisiana (LA), New York (NY), and Oklahoma (OK), and Pennsylvania (PA). Ticks from Covington, Louisiana [30.47671° N, −90.10517° E]; Stillwater, Oklahoma [36.110176° N, −97.05857° E]; and State College, Pennsylvania [40.790703° N, −77.858795° E], were collected in spring 2019, and ticks from Millbrook, New York [41.785538° N, −73.69046° E] were collected in winter of 2019. Unfed adult *Ix. scapularis* ticks mate before attachment on a vertebrate host. Ticks were identified by using standard morphological keys. To test the microbes’ transfer during mating of male and female, unfed ticks (♂ and ♀) were used for these experiments. Unmated female and male ticks were kept in a vial for 48 h and allowed to mate with their partners. Unfed adult ticks from Pennsylvania were blood-fed, as described earlier [61], and tissues were dissected from partially engorged female ticks. The dissecting solution was ice cold 100 mM 3-(N-Morpholino-propanesulfonic acid (MOPS) buffer containing 20 mM ethylene glycol bis-(β-aminoethyl ether)-N, N, N’, N’-tetraacetic acid (EGTA), pH 6.8. After removal, salivary glands, midgut, and ovaries were washed gently in the same ice-cold buffer. The dissected tissues were stored immediately after dissection in DNA lysis buffer before isolating DNA. The experimental plan for this study is illustrated in Figure 10. Briefly, at least three biological replicates were used for each biological sample (unfed and partially engorged ticks) and tissue types (midgut, salivary glands, and ovary) from Pennsylvania ticks. Additionally, three biological replicates for each of dissected tissues and whole tick were used in this study. The unfed ovary tissues were excluded from Pennsylvania in these analyses due to the small size and technical challenge in getting enough DNA from a single dissected ovary. Partially engorged ovaries from Pennsylvania ticks were dissected for these experiments.

### 4.2. DNA Isolation

Genomic DNA was extracted from (1) individual whole unfed ticks from Louisiana, New York, Oklahoma, and Pennsylvania; and (2) unfed and partially engorged tick tissues (salivary gland, midgut, ovary) from Pennsylvania, only with the DNeasy Blood and Tissue kit catalog # 69506 (Qiagen, Valencia, CA, USA), using the standard protocol provided by the manufacturer. Before processing, the ticks were sterilized by two rounds of subsequent washing in 10% bleach, 70% ethanol, and sterile phosphate-buffered saline (PBS) (Biosciences, Cat#R028, St. Louis, MO, USA). DNA concentration and purity were analyzed by using Nanodrop, and the extracted DNA samples were stored at −20 °C until further use.

### 4.3. Illumina Library Preparation and 16S rRNA Sequencing

Illumina DNA library preparation and 16S sequencing were performed by MR DNA, Shallowater, TX, USA. V1–V3 variable region of 16S rRNA genes was amplified using the forward primer 27F (5′-GAGTTTGATCNTGGCTCAG-3′) and the reverse 519R (5′-GTNTTACNGCGGCKGCTG-3′) with a barcode on the forward primer. The HotStarTaq Plus Master Mix Kit (Qiagen, USA) was used with the following PCR conditions: −94 °C for 3 min, followed by 30 cycles of 94 °C for 30 s, 53 °C for 40 s, and 72 °C for 1 min, after which a final elongation step at 72 °C for 5 min was performed. Amplified products were checked on 2% agarose gel to confirm the appropriate size and intensity of bands. On the basis of molecular weight and DNA concentrations, equal proportions of multiple samples were pulled together and purified by using calibrated Ampure XP beads. Size of the DNA amplicons was determined by running on 2% agarose gel. Expected size of the DNA band was ~500 bps. Each sample was diluted to 5 nM, and 5 µLs of each sample was added to the pool. The quality and size of the DNA libraries was confirmed by lab-on-chip analysis, using the Bioanalyzer (Agilent Technologies, Inc., Santa Clara, CA, USA). The pooled sample was sent for sequencing. The mathematical formula used to convert ng/µL to nM is as follows:

$$\frac{\left(concentration\;in\;\mathrm{ng}/\mu L\right)}{\left(660\;g/\mathrm{mol}\;x\;average\;library\;size\right)}\times 10^{6}=concentration\;in\;\mathrm{nM}$$

The average molecular mass of one base-pair DNA is 660 g/mol. 

Purified PCR products were used to prepare Illumina DNA library. The quality of the DNA libraries was confirmed by lab-on-chip analysis, using the Bioanalyzer (Agilent Technologies, Inc., Santa Clara, CA, USA), and then 16S sequencing was performed on Illumina MiSeq platform at MR DNA, Shallowater, TX, USA. Three biological replicates of each of the controls were used. Controls used were DNA extraction blank control, negative control (buffer), negative control (sterile water), no-template control, and positive-DNA extraction control [62,63] (commercially available Mock Microbial Community Standard, ZymoBIOMICS catalog # D6306).

### 4.4. Data Processing

Quantitative Insights into Microbial Ecology (QIIME 2, https://qiime2.org accessed on 13 February 2021) was used for sequence analysis. Raw fastq files were processed by fastq processor available on the MR DNA website, which provided the files compatible with the Earth microbiome project (EMP) paired-end format. Then “Atacama soil microbiome” tutorial (website link: https://docs.qiime2.org/2021.2/tutorials/atacama-soils/ (accessed on 13 February 2021)) and “moving pictures” tutorial (website link: https://docs.qiime2.org/2021.2/tutorials/moving-pictures/ (accessed on 13 February 2021) were followed to process the sequencing data. Among whole-tick samples, the maximum number of reads for a sample was 114,478, while the minimum number of reads for a sample was 36,358. For tick tissue samples, the maximum number of reads for a sample was 56,785, while the minimum number of reads was 17,258. The abovementioned ranges of sequences of each sample type were available before the denoising step (DADA2 processing). DADA2 [64] was used for trimming, primer sequence removal, sequence denoising, paired-end merging, filtering of chimeric sequences, singleton removal, and sequence dereplication. This step yielded 5000 sequences from each tissue sample and 16,000 sequences from each of the whole-tick samples for rarefaction curves. The rarefaction curve is getting leveled out, suggesting that collecting additional sequences beyond that sampling depth would not observe additional reads. Minimum overlap of 50 bases was used for paired-end merging. Resultant sequences sets obtained after DADA2 processing were aligned by MAFFT (ver.7) [65], and then a phylogenetic tree was created by using FastTree (ver. 2.1) [66]. Greengenes 13_8 99% OTU database [67] was used to train the Naïve Bayes classifier, to which the represented sequences were compared and a 97% sequence similarity was put as a cutoff for taxonomic classification. Network correlation maps were inferred based on the Sparse Correlations for Compositional data (SparCC) approach [68]. This approach uses the log-transformed values to carry out multiple iterations and subsequently identify taxa outliers to the correlation parameters [69]. Raw sequences were submitted to the NCBI read under the SRA database and obtained the accession number PRJNA663181.

### 4.5. Statistical Analysis

To measure α-diversity, different indices, such as Faith’s phylogenetic diversity (faith_pd) and Pielou’s community evenness (pielou_e), were used. Faith’s phylogenetic diversity (faith_pd) is an unweighted measure of phylogenetic distance of observed sequences; and Pielou’s community evenness (pielou_e) measures how evenly bacterial species are distributed within a community. Kruskal–Wallis non-parametric tests (*p* ≤ 0.05) were performed to determine statistical significance of alpha-diversity metrics by using QIIME 2. Weighted and unweighted UniFrac Metrics [70] were used for β-diversity analysis. EMPeror [71] was used for visualization of principal coordinate analysis (PCoA) plot, and PERMANOVA tests (*p* ≤ 0.05) were used to test the statistical significance of β-diversity measurements.

## 5. Conclusions

This study has shown significant differences in the microbiome of *Ix. scapularis* ticks collected from Northeastern (New York and Pennsylvania) and Southern (Oklahoma and Louisiana) states. These results provide an insight into the microbiome of New York, Pennsylvania, Oklahoma, and Louisiana tick populations and the interplay between pathogenic and endosymbiotic rickettsiae. The question remains: what are the drivers behind these variations among the microbiome composition and diversity? This question warrants further investigation into issues such as why Oklahoma ticks contain comparatively much higher level of Firmicutes, while ticks from all other locations included in this study contain almost negligible Firmicutes. In further analysis at the species level, it was revealed that these Firmicutes from Oklahoma ticks possess bacterial species, such as *Jeotgalicoccus* sp. and *Staphylococcus lentus*. Do these species restrict *B. burgdorferi* s.l. in Oklahoma ticks? Similarly, the level of Bacteroidetes is much higher in Northeastern ticks when compared to Southern ticks, whose levels are almost insignificant. Further analysis revealed that these Bacteroidetes possess bacterial species, such as *Sphingobacterium faecium*, *Flavobacterium* sp., *Sphingobacterium* sp., *Flexibacter* sp., and *Flavobacter* spp. Do these Bacteroidetes interact with spirochetes to colonize Lyme disease causative agent (i.e., *B. burgdorferi* s.l.) into Northeastern ticks? Probable competitive interaction between *R. buchneri* and *B. burgdorferi* s.l. surfaced in this study and is subject to be further investigated by using dysbiosis experiments and a bigger sample size.

## Data Availability

Data supporting the conclusions of this article are included within the article and its additional files. The raw datasets used and analyzed for the present study are available from the corresponding author upon reasonable request.

## Supplementary Materials

The following supporting information can be downloaded at. https://www.mdpi.com/article/10.3390/pathogens11020130/s1. Figure S1: Rarefaction Curve. Table S1: Comparison of bacterial profiling and relative abundance (phylum level) between the ticks collected from Northeastern and Southern regions of the United States. Table S2: Comparison of bacterial profiling and relative abundance (species level) between the ticks collected from Northeastern and Southern regions of the United States. Table S3: Comparison of bacterial profiling and relative abundance (phylum level) in unfed (UF) and partially fed (PF) tick tissues collected from Pennsylvania. Table S4: Relative abundance of Borrelia burgdorferi and Rickettsia buchneri in tick tissues from Pennsylvania (PA). Table S5: Absolute abundance (mean of number of sequences of biological replicates) of Borrelia burgdorferi and Rickettsia buchneri in tick tissues from Pennsylvania (PA). Table S6: Unweighted-Unifrac-pairwise PERMANOVA analysis (beta-diversity) for whole tick samples collected from Louisiana (LA), Oklahoma (OK), Pennsylvania and New York (NY). Table S7: Network correlation summary across all datasets. Table S8: Network correlation summary showing significant interactions between ticks from Pennsylvania. Table S9: Network correlation summary showing significant interactions between ticks from Louisiana. Table S10: Network correlation summary showing significant interactions between ticks from Oklahoma.
